# Friend or Foe: Whole-Genome Sequence of Pseudomonas aeruginosa TG523, Isolated from the Gut of a Healthy Nile Tilapia (Oreochromis niloticus)

**DOI:** 10.1128/mra.01133-22

**Published:** 2023-01-04

**Authors:** Sulav Indra Paul, Ashikur Rahman, Md Mahbubur Rahman

**Affiliations:** a Institute of Biotechnology and Genetic Engineering, Bangabandhu Sheikh Mujibur Rahman Agricultural University, Gazipur, Bangladesh; University of Arizona

## Abstract

Here, we present the genomic features of Pseudomonas aeruginosa TG523, which was isolated from the gut of a healthy Nile tilapia (Oreochromis niloticus). With a genome size of 6,381,902 bp with 5,931 open reading frames, the genome harbored genes predicted to have antibacterial activity and those which are implicated in virulence.

## ANNOUNCEMENT

**FIG 1:**
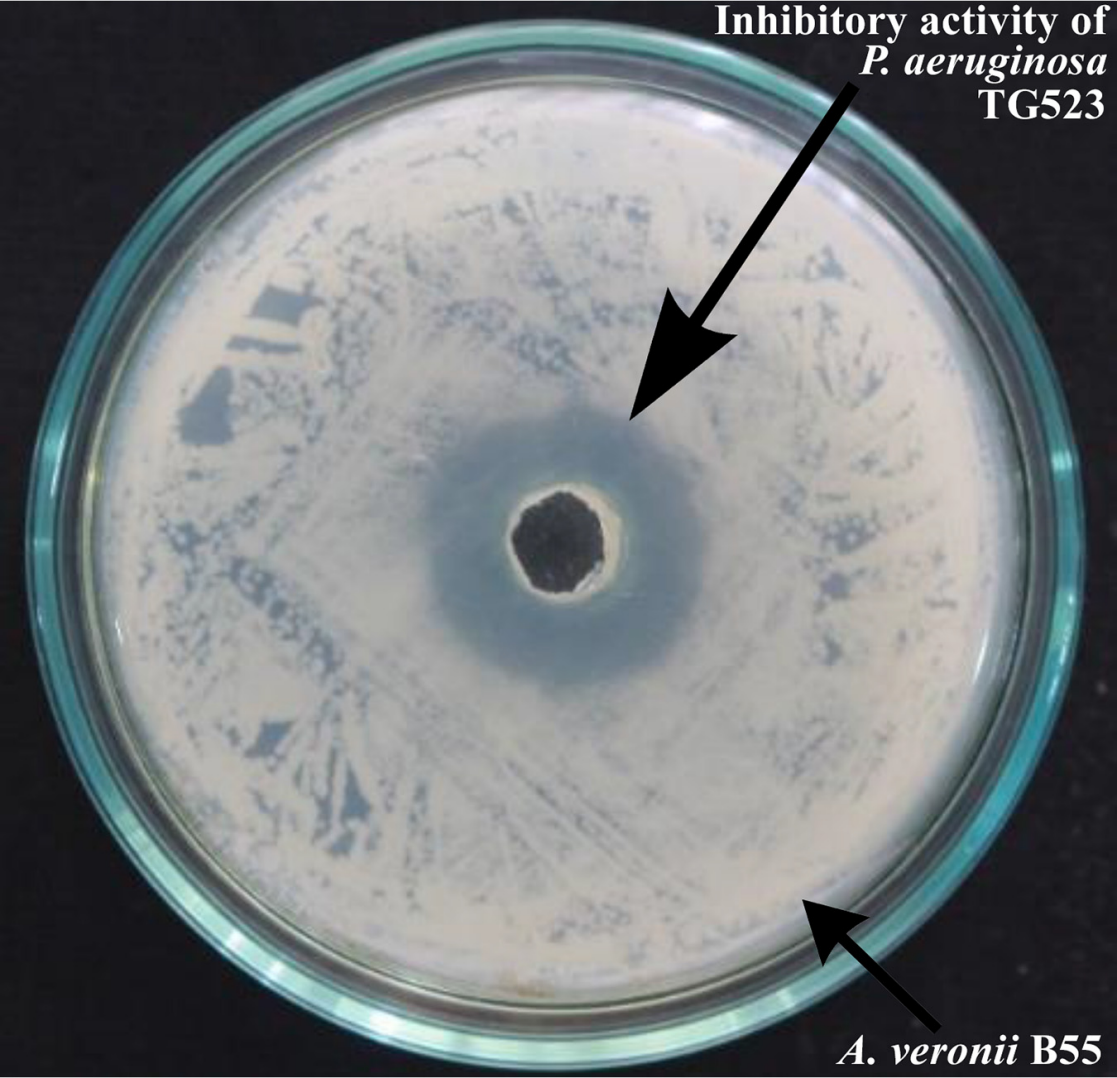
*In vitro* inhibition zone of the cell-free culture supernatant of P. aeruginosa TG523 against a highly virulent fish pathogenic strain of *A. veronii*, B55.

Pseudomonas aeruginosa strains can produce a variety of secondary metabolites and biopolymers with bactericidal or bacteriostatic activities (1), but they can also be used as a probiotic in aquaculture (2–4). To isolate strain TG523, the abdomen of a healthy Nile tilapia was cut aseptically, and the gut was taken out. One-gram homogenates of the intestinal segments were serially diluted and spread onto de Man, Rogosa, and Sharpe (MRS) agar plates and incubated at 28°C for 48 h. TG523 was picked from the growing colonies on the MRS plate. The antimicrobial activity of TG523 was determined by an agar well diffusion assay as described previously (5). Briefly, TG523 was grown in MRS broth at 28°C for 7 days. The culture was centrifuged at 10,000 × *g* for 15 min, and the culture supernatant was passed through a Millipore membrane filter for an *in vitro* inhibition assay against fish pathogens. The cell-free culture supernatant of TG523 exhibited *in vitro* inhibitory activities against a highly virulent fish pathogen, Aeromonas veronii B55 (Fig. 1). This bacterium also exhibits *in vivo* growth promotion and suppresses motile *Aeromonas* septicemia (MAS) in Nile tilapia (6). The animal experiments obtained ethics approval from the Institute of Biotechnology and Genetic Engineering (IBGE) ethical review committee (approval number IBGE-ERC-008).

A single colony of TG523 was inoculated in MRS broth and incubated at 28°C for 48 h. Then, the DNA was extracted from the MRS broth using a GeneJET genomic DNA purification kit (Thermo Fisher Scientific, USA) according to the manufacturer’s instructions. Extracted DNA was quantified using a NanoDrop spectrophotometer (Thermo Fisher Scientific). The 16S rRNA gene was amplified using the primer pair 8F (5′-AGAGTTTGATCCTGGCTCAG-3′) and 1492R (5′-GGTTACCTTGTTACGACTT-3′), which exhibited 99.93% homology (identities 1356/1357) with the P. aeruginosa strain DSM 50071. A paired-end DNA library was prepared using the same DNA used for 16S rRNA sequencing using a Nextera XT library prep kit (Illumina, San Diego, CA) according to the manufacturer’s instructions (7). Genome sequencing (600 cycles) was carried out using the MiSeq benchtop sequencer (Illumina) (8, 9), yielding a total of 5,258,026 reads (2,629,013 paired-end reads) and 1,343,253,926 bases. PRINSEQ v.0.20.3 (10) was used to assess the quality of the reads. Trimmomatic v.0.38 (11) was used for trimming low-quality sequences. The *de novo* assembly was performed using quality reads into draft genomes using SPAdes v.3.9.0 (12) and resulted in 68 contigs, a total length of 6,381,902 bp, an *N*_50_ value of 297,349 bp, a G+C content of 66.43%, and genome coverage of 210×. The largest and smallest contigs were 606,150 bp and 501 bp, respectively. QUAST v.5.0.2 (13) was used for the quality assessment of the assembled genome. Genome annotation was performed using the NCBI Prokaryotic Genome Annotation Pipeline (PGAP) (https://www.ncbi.nlm.nih.gov/genome/annotation_prok/) (14) and identified 5,931 total coding sequences (CDSs) with 5,881 CDSs encoding putative proteins, 68 RNA genes (58 tRNAs, 6 rRNAs, and 4 noncoding RNAs), and 50 pseudogenes. Using SpeciesFinder v.2.0 (https://cge.food.dtu.dk/services/SpeciesFinder/) (15), the bacterium was identified as P. aeruginosa. Secondary metabolite biosynthetic gene clusters (SM-BGCs) were identified using antiSMASH v.6.0 (16). Default parameters were used for all bioinformatics analyses except where otherwise noted.

RAST v.2.0 (17) predicted 390 subsystems and 2,527 protein-coding genes that were determined to belong to putative functional categories. antiSMASH v.6.0 (16) detected several SM-BGCs, including pyoverdine, pyocyanin, lankacidin-C, oxalomycin-B, streptophenazines, bicyclomycin, pyochelin, and l-2-amino-4-methoxy-*trans*-3-butenoic acid. A siderophore, pyoverdine, is a virulence factor having iron-gathering capacities resulting in the damage of host mitochondria (18). PathogenFinder v.1.1 (https://cge.food.dtu.dk/services/PathogenFinder/) (19) predicted TG523 as a human pathogen (matched 594 pathogenic families). On the contrary, the secondary metabolites pyocyanin, lankacidins, and streptophenazines are well-known for their antibacterial actions against various pathogens (20–22). It remains to be determined whether P. aeruginosa TG523 is among our beneficial microbial friends or pathogenic foes, given its possession of both antibacterial traits, *in vivo* growth promotion and disease prevention in Nile tilapia, as well as the presence of virulence genes in its genome.

### Data availability.

The whole-genome shotgun project of Pseudomonas aeruginosa TG523 has been deposited at GenBank under the accession number JANTNS000000000. The associated BioProject and BioSample accession numbers are PRJNA870908 and SAMN30388467, respectively. The raw data are available from the Sequence Read Archive (SRA) under accession number SRX17158524. The 16S rRNA gene sequence has been deposited at GenBank under the accession number MW512507.
